# Physiological Dose of EGCG Attenuates the Health Defects of High Dose by Regulating MEMO-1 in *Caenorhabditis elegans*

**DOI:** 10.1155/2021/5546493

**Published:** 2021-06-24

**Authors:** Yan Lu, Yi Wang, Li-gui Xiong, Jian-an Huang, Zhong-hua Liu, Yu-shun Gong

**Affiliations:** ^1^National Research Center of Engineering and Technology for Utilization of Botanical Functional Ingredients from Botanicals, Hunan Agricultural University, Changsha, Hunan 410128, China; ^2^Key Laboratory of Tea Science of Ministry of Education, Hunan Agricultural University, Changsha, Hunan 410128, China; ^3^Collaborative Innovation Centre of Utilization of Functional Ingredients from Botanicals, Hunan Agricultural University, Changsha, Hunan 410128, China

## Abstract

EGCG, as a dietary-derived antioxidant, has been extensively studied for its beneficial health effects. Nevertheless, it induces the transient increase in ROS and leads to the hormetic extension of lifespan. How exactly biology-benefiting effects with the minimum severe adverse are realized remains unclear. Here, we showed that physiological dose of EGCG could help moderate remission in health side effects exposed to high doses, including shortened lifespan, reduced body size, decreased pharyngeal pumping rate, and dysfunctional body movement in *C. elegans.* Furthermore, we found this result was caused by the physiological dose of EGCG to block the continued ROS accumulation and triggered acclimation responses after stressor removal. Also, in this process, we observed that EGCG downregulated the key redox protein MEMO-1 to activate the feedback loop of NADPH oxidase-mediated redox signaling. Our data indicates that the feedback signal induced by NADPH oxidase may contribute to the health-protective mechanism of dietary polyphenols *in vivo*.

## 1. Introduction

Phytochemicals are secondary plant metabolites found in human diets, also known as “phytonutrients” [1]. It is believed to be a matter of health benefits, due to its content of polyphenols, vitamins, fiber, phytosterols, and carotenoids [2–4]. Epigallocatechin-3-gallate (EGCG) is the most abundant dietary polyphenolic derived from green tea [*Camellia sinensis L. Ktze.* (*Theaceae*)], which has been shown to participate in the regulation of various metabolic processes and has been suggested as therapeutic health effects for a variety of chronic pathological conditions, such as cancer, obesity, diabetes, cardiovascular diseases, and neurodegenerative diseases [2, 5–8].

To date, EGCG as a well-recognized antioxidant in food chemistry is frequently used in the public press and by the dietary-supplement industry to imply beneficial health effects. Earlier studies suggested that the intrinsic antioxidant properties may be the sole reason for its health benefits [9–13]. Indeed, it has been proven to be directly related to prevent oxidative damage and mediate life expedition in diverse model organisms, including Cells [14], *C. elegans* [15, 16], fruit fly [17], rat [18–20], mice [21], and human [22]. Nevertheless, recent indications suggested that the sweeping statement of an antioxidative capacity of EGCG is no longer able to explain beneficial effects [4]. It was pointed towards that there is no single universal antioxidant equated to health, which may be an antioxidative independent or indeed prooxidant action [23, 24]. Such prooxidant effects appeared to be responsible to play an important role in antibacterial, antiobesity, and other chronic disease prevention and treatment by interfering with many biochemical processes [6, 25–31]. Meanwhile, the prooxidant property of EGCG has been further demonstrated in terms of cancer prevention and healthy lifespan extension through inducing endogenous antioxidant systems and trigging the mitohormesis. This was later considered to be a potential mechanism for the health benefits of EGCG [29, 32–34].

In spite of these generally beneficial effects, high doses of EGCG not only caused cytotoxicity *in vitro* but also resulted in living body hepatotoxicity, nephrotoxicity, and gastrointestinal disorders (vomiting and diarrhea) [35, 36]. Taken together, existing literature concerning the health effects of EGCG reveals that the antioxidant/prooxidant mechanism is vague, which cannot explain, in full, the magnitude of impact remains elusive. Here, we used the model organism *C. elegans* with obvious health indicators to elucidate the health-protective mechanism by which EGCG modulated physiological health indicators, in both prooxidant and antioxidant ways, and to further investigate how physiological doses of EGCG to response to oxidative stress exposed to high doses. We reported that a physiological dose of EGCG could induce moderate remission in health defects exposed to high doses, such as shortened lifespan, reduced body size, decreased pharyngeal pumping rate, and body movement dysfunction. This was achieved either by scavenging ROS through physiological doses of antioxidants or by inducing a moderate increase in stress adaptation through prooxidant effects. These findings revealed that the physiological dose of EGCG that can alleviate health deficits caused by high-dose exposure through regulating the NADPH oxidase-mediated redox signaling with MEMO-1 as a key core.

## 2. Materials and Methods

### 2.1. Nematode Strains and Culture Conditions

Standard conditions for *C. elegans* strains culture and all experiments were performed at 20°C on nematode growth media (NGM) agar supplemented with *Escherichia coli* (OP50), unless otherwise indicated. The following strains which were obtained from the Caenorhabditis Genetics Center (CGC) were used in this study: N2—wild-type Bristol isolate, VC794—*memo-1*(*gk345*), CB767—*bli-3*(*e767*), and QZ50—jxEx8[P*memo-1*::GFP; P*myo-3*::RFP]. In addition, VC794: *memo-1*(*gk345*); CB767: *bli-3*(*e767*); and QZ50: jxEx8[P*memo-1*::GFP; P*myo-3*::RFP] were provided by the Prof. Dr. Collin Y. Ewald (ETH Zurich) [37, 38].

### 2.2. *E. coli* Strains

The monoclonal bacteria of E. coli OP50 was inoculated into LB to be incubated overnight at 37°C with shaking and then expanded cultivation for 4 h. Concentrated *E. coli* was resuspended with M9 and directly coated onto NGM for experiment.

### 2.3. Chemicals and Their Treatment

EGCG (98% pure) was purchased from Sigma-Aldrich (St. Louis, MO, USA) and stored in water solution at -20°C, which were added into NGM media from a high concentration stock solution before pouring of the plates. Antioxidants were added with *E. coli OP50* on the experimental NGM plate and allowed to dry (200 *μ*M, DL-dithiothreitol (DTT); 2 mM, N-acetyl-L-cysteine (NAC); 5 mM, ascorbic acid (VC)). Plates were stored at 4°C and made fresh each week. Synchronization larva were transferred from normal NGM plates to drug plates for experiment. For all experiments, stock solutions were freshly prepared in distilled water, unless specified, and sterilized by filtration through 0.2 *μ*m pore size membranes prior to administration.

Larvae of *wild-type* or mutants which have been synchronized (L1 stage) were subjected to intervention in the control and treatment groups (2 mM EGCG, 0.2 mM EGCG, and 2 mM EGCG +antioxidants) for 72 h. Then, the poststress worms were transferred to the experimental dishes in the therapeutical group, and health indicators were measured and observed on days 0, 2, 4, and 6 of adulthood.

### 2.4. Body Size Measurement of *C. elegans*

Body length was defined from the buccal cavity to the end of the hindgut. Body width was defined in terms of gonad position. Body-length measurements were carried out by acquiring digital images at 5x magnification on a ZEN microscope of more than 10 larvae and adults per condition. The body length and body width of the imaged *C. elegans* were traced with short line segments using ZEN3.1 blue software and calculated according to equation (1). Each experiment was performed at least twice.

(1) $$\begin{array}{c} \text{Estimated body size}\left(\times \frac{10^{6}\mu {\text{m}}^{3}}{\text{worm}}\right)=\pi {\left(\frac{\text{body width}}{2}\right)}^{2}\times \text{body length}. \end{array}$$

### 2.5. Pharyngeal Pumping Rate

At the 0, 2rd, 4th, and 6th days of adulthood, the pharyngeal pumping rate was quantified. In detail, for each concentration and time point, 6 (pretreated) F1 nematodes were randomly selected, and the pumping frequency was determined three times every minute, each over a time span of 15 s.

### 2.6. Lifespan Experiments

Lifespan assays were performed according to standard protocols unless otherwise indicated. In brief, at the preferred tile larva stage one (L1), age-synchronized nematodes were transferred to NGM plates with different concentration of EGCG, and it was defined as day 0. Surviving and dead animals were counted every two days until all individuals had died. Worms that failed to respond to a gentle touch were scored as dead. All lifespan experiments were conducted in a double-blind manner. The SPSS 23.0 (Demo version, Armonk, NY, USA) statistical analysis package was used for all lifespan. Lifespan assay experiments were analyzed using the Kaplan-Meir test, and *P* values were calculated with the log-rank test.

### 2.7. Measuring Total ROS Levels with Fluorescent Probe

The total level of ROS was measured by using a small diffusible fluorescent probe (dihydroethidium, DHE, Invitrogen, MA, 5*μ*М; Mitosox, Invitrogen, MA, 5*μ*М). More than 20 animals per conditions were harvested into asepsis pipes and incubated in the dark for 1 h. And then worms were mounted on a thick layer of half-dried agar pad on microscopic glass slides to measure the fluorescent intensity with confocal microscopy (Zeiss LSM 710) and analyzed by Zeiss 3.1 blue imaging software.

### 2.8. Confocal Microscopy and Image Processing

For confocal imaging of fluorescence probe, the transgene strains were immobilized with 4 mM solution of tetramisole hydrochloride in M9 and mounted on 6% agarose pads on glass slides. Worm imaging was carried out using a Zeiss LSM 710 inverted confocal microscope (Carl Zeiss AG, Jena, Germany) under nonsaturating exposure conditions. For each condition, multiple worms were observed and imaged. Image processing was performed using Zeiss 3.1 blue software.

### 2.9. Measurements of ATP Levels

ATP levels in whole worms were determined as described [39]. For larval development experiments, approximately 4,000–5,000 worms were age-synchronized using the double-bleaching method transferred to NGM agar surface (16-cm diameter) containing EGCG that was fully covered with *E. coli* OP50 and grown at 20°C. The larvae were washed off the agar surface using a pipette filled with M9 buffer and repeatedly resuspended to remove *E. coli*. Worms were collected every 24 hours during the development stage after feeding developmentally arrested L1 animals. For adult growth experiments, 40-50 adults per plate were picked into EP tube every 24 hours after transferring to the remission experimental plates at a low-dose EGCG or antioxidant treatment. Protein concentrations were normalized to total protein content as determined by a Micro BCA protein assay kit (Thermo Fisher, Cat #23235). ATP was measured in technical triplicates, and the average ATP concentration per *μ*g protein was calculated per biological sample with at least 3 biological experiments for each time point unless indicated otherwise.

### 2.10. Statistical Analysis

All results were presented as the mean ± SEM. Statistical significance was assessed using ANOVA or Student's *t*-test when appropriate, and *P* < 0.05 was considered statistically significant.

## 3. Results

### 3.1. Physiologic Doses of EGCG Can Improve the Lifespan of *C. elegans* Exposed to High Dose

**Figure 1:**
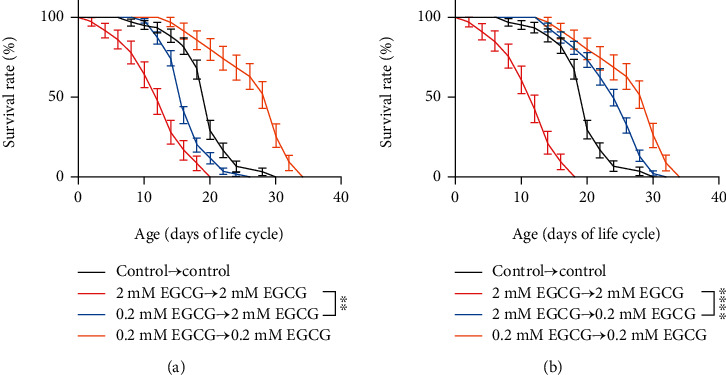
Physiologic dose of EGCG prolongs the lifespan of *C. elegans* exposed to high dose. (a) Survival rate changes in the lifespan of N2 worms, where the larvae was treated with physiologic doses of EGCG and then was treated with high doses of EGCG during adulthood. ^∗∗∗^*p* < 0.001, by two-way ANOVA. (b) Survival rate changes in the lifespan of N2 worms, where the larvae was treated with high dose of EGCG and then was treated with physiologic doses of EGCG during adulthood. ^∗∗∗^*p* < 0.001, by two-way ANOVA.

Based on the results of our previous studies that EGCG extended the lifespan of adult *C. elegans* in an inverted U-shaped dose-response manner, here, we treated with 2 mM EGCG (high dose EGCG) and 0.2 mM EGCG (physiologic doses EGCG) on the synchronized larvae, respectively. It showed that there still was a hormesis effect of the concentration of EGCG on longevity, even if the intervention started from larval development (Figure 1(a)). We then transferred/picked the larvae that were intervened for 72 h by high dose or physiologic doses to the fresh NGM plates containing physiologic doses or high dose correspondingly, to assess the lifespan of adults during poststress growth. The comparison revealed that after the high dose of EGCG was halted, the shortened lifespan of *C. elegans* was prolonged by the physiologic doses of EGCG. Surprisingly, the lifespan of the 2 mM EGCG→0.2 mM EGCG group was almost extended by the inclusion of physiological doses of EGCG in agar. In such case, it still can see a similar phenomenon for moderate poststress larvae that was transferred to higher doses of EGCG (Figures 1(a) and 1(b)). Thus, EGCG-mediated lifespan in *C. elegans* depends on its concentration and period of intervention.

### 3.2. Physiologic Doses of EGCG Can Improve the Development of the Body Size, Pharyngeal Pumping, and Movement of *C. elegans* Exposed to High Dose

**Figure 2:**
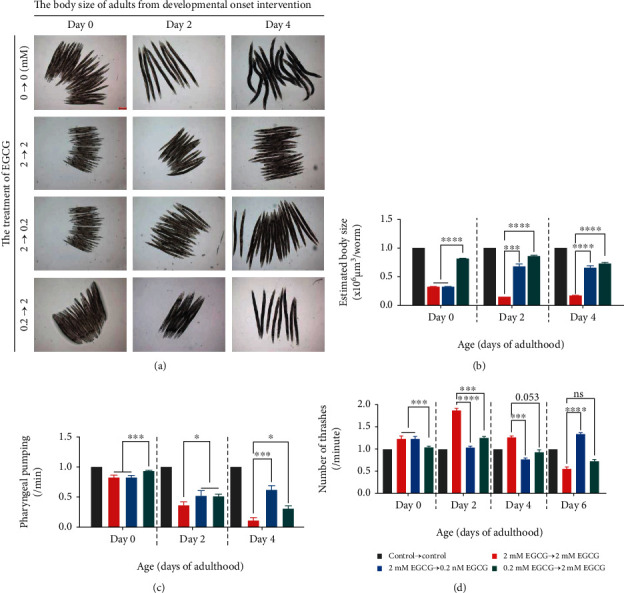
Physiologic dose of EGCG improves healthy physical indicators of *C. elegans* exposed to high dose. (a) The body size of adults from development onset intervention with physiologic doses and high doses of EGCG. Scale bar: 200 *μ*m. (b) Estimation body size per worm, three replicate experiments; *n* ≥ 6 worms. ^∗^*p* < 0.05, ^∗∗^*p* < 0.01, and ^∗∗∗^*p* < 0.001, by two-way ANOVA. (c) Pharyngeal pumping rate per min on plates, three replicate experiments; *n* ≥ 10 worms. ^∗^*p* < 0.05, ^∗∗^*p* < 0.01, and ^∗∗∗^*p* < 0.001, by two-way ANOVA. (d) Number of thrashes per minute in liquid. Three replicate experiments; *n* ≥ 10 worms. ^∗^*p* < 0.05, ^∗∗^*p* < 0.01, and ^∗∗∗^*p* < 0.001, by two-way ANOVA.

Given the related changes of neuromuscular behaviors and morphology of *C. elegans* during aging, we set out to verify whether EGCG caused the same effect on worm activity with the lifespan following the same treatment described above to measure the pharyngeal pumping rate, body movement, and body size [40]. These studies revealed that the worms cultivated with the high dose EGCG continuously from the larval development were significantly reduced both in body size and pharyngeal pumping rate during aging as compared with control, while physiological doses did not interfere with pharyngeal pumping and body movement, except for a little reduction in body size (Figures 2(a)–2(c)).

Interestingly, the body movement of larvae during early intervention of EGCG showed a brief rise followed by a sharp decline and even partial paralysis, unlike the control (Figure 2(d) and Figure S1), whereas, regardless of whether the intervention is for larvae or adults, as expected, these health defects were significantly alleviated by at least 24 hours following physiological dose treatment, as shown in body size and pharyngeal pumping parameters. Similarly, the symptom of paralysis was relieved when the high dose was removed and replaced with a physiological dose of EGCG, which took almost 6 days (Figure 2(d)). Moreover, we also found that unlike other intervention groups, the worms of the 0.2 mM EGCG→2 mM EGCG group developed a decline in both neuromuscular behaviors and morphology after day 4 of adulthood as compared to the control and did not regain the body movement from day 6 of adulthood (Figure 2(d)). Thus, physiological doses of EGCG that have been shown to extend healthy lifespan can alleviate the potential side effects of the high dose in health-related physiological indicators through yet undetermined biological activity.

### 3.3. EGCG Exerts Its Biochemical Function through Regulating ROS

**Figure 3:**
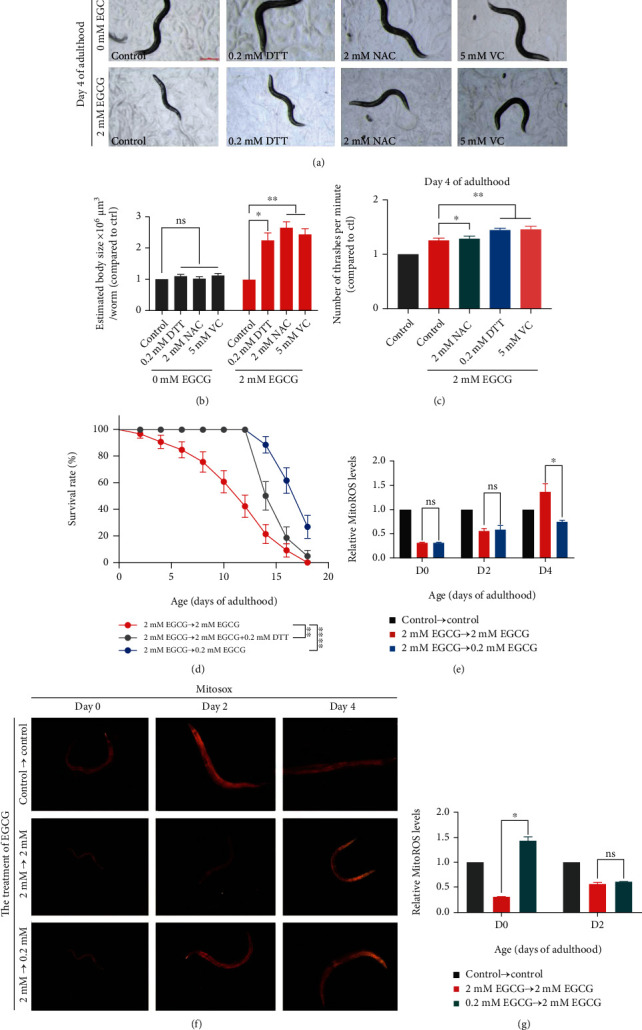
EGCG exerts its biochemical function through regulating ROS. (a) Antioxidants mitigate health indicator of *C. elegans* after high-dose EGCG stress at day 4 of adulthood. Scale bar: 200 *μ*m. (b) Estimation body size per worm treated with antioxidants, three replicate experiments; *n* ≥ 6 worms. ^∗^*p* < 0.05, ^∗∗^*p* < 0.01, and ^∗∗∗^*p* < 0.001, by two-way ANOVA. (c) Number of thrashes per minute in liquid. Three replicate experiments; *n* ≥ 10 worms. ^∗^*p* < 0.05, ^∗∗^*p* < 0.01, and ^∗∗∗^*p* < 0.001, by two-way ANOVA. (d) Survival rate changes in the lifespan of N2 worms, where the larvae were treated with high dose of EGCG and then was treated with high dose of EGCG combined with DTT during adulthood. ^∗∗∗^*p* < 0.001, by two-way ANOVA. (e) Relative quantification of Mitosox of worm treatment with EGCG at day 0, day 2, and day 4. ^∗^*p* < 0.05, by two-way ANOVA. (f) Representative images of Mitosox reporter worms treated with vehicle or EGCG, showing quality of mitochondria ROS following. Scale bar: 200 *μ*m. (g) Relative quantification of Mitosox of worm treatment with EGCG at day 0 and day 2 of adulthood, where the larvae are treated with physiologic doses of EGCG and then was treated with high doses of EGCG during adulthood. ^∗^*p* < 0.05, by two-way ANOVA.

To further explore the biological activity and mechanism of action of EGCG *in vivo*, we selected the ROS-specific fluorescent dye DHE for staining. We found that high doses of EGCG intervention 48 hours in larvae appeared to promote a surge in ROS, which was decreased to normal levels at the early stages of young adulthood (Figure S2a and S2b). Next, we transferred the poststress larvae to a fresh plate containing the chemical antioxidant (200 *μ*M, DL-dithiothreitol (DTT); 2 mM, N-acetyl-L-cysteine (NAC); 5 mM, ascorbic acid (VC)) and high dose of EGCG for the simultaneous intervention. A slight alleviation in the reduction of body size and body paralysis of *C. elegans* has occurred when either DTT, NAC, or VC added to the plate, respectively (Figures 3(a)–3(c)). In such cases, DTT, often used as an antioxidant to scavenge mitochondrial ROS, has also been found to improve the lifespan of high-dose EGCG interventions (Figure 3(d)). Moreover, the experimental data for the mitochondrial ROS measured by MitoSOX also supported that a high dose of EGCG induced more accumulation of ROS than that of control, while ROS accumulation became less after 4 days of physiological dose treatment during adulthood (Figures 3(e) and 3(f)). Whereas, in the experiments described above, we tested the mitochondria ROS at the end of larval development and found that it remained high in the physiological dose treatment group, the high dose of EGCG puts mitochondrial ROS in a period of buffered decline, which may be a result of the oxidative stress response (Figure 3(g)). These results suggested that the recovery treatment of physiological doses on poststress larvae can moderately induce the antioxidant system to efficiently counteract ROS to render physiological index healthy that would be otherwise dysfunction in a sustained high-dose stress condition. Taken together, these findings supported that physiological doses of EGCG enabled ROS to detoxify through health protection mechanism, which served to reduce the levels of ROS and potentially contributed to mitigate the health defects.

### 3.4. EGCG Activates NADPH Redox Feedback Signaling through Moderate Inhibition of MEMO-1

**Figure 4:**
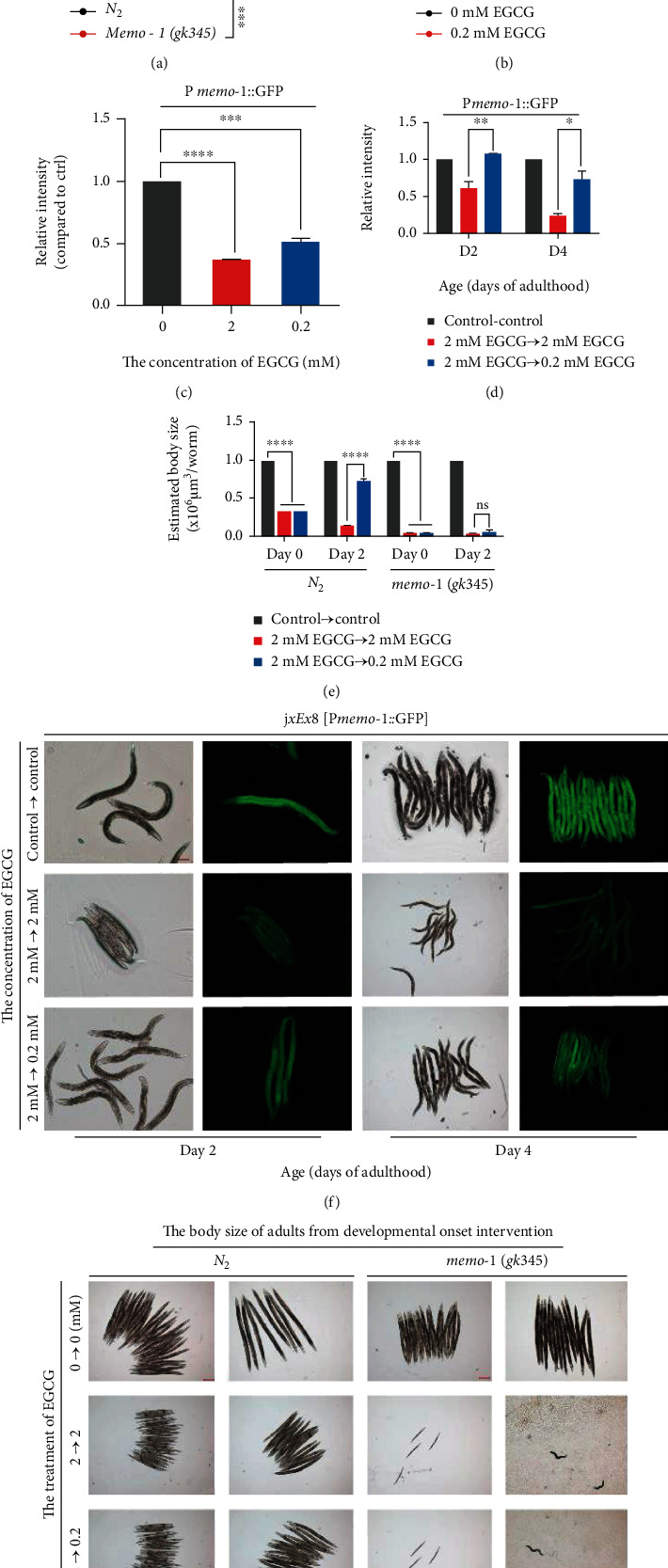
EGCG activates NADPH redox feedback signaling through moderate inhibition of MEMO-1. (a) Survival rate changes in the lifespan of EGCG extended lifespan in the *memo-1(gk345)* mutants. (b) Survival rate changes in the lifespan of EGCG extended lifespan in the *bli-3(e767)* mutants. (c) Relative quantification of P*memo-1*::GFP of worm treatment with EGCG at day 0. ^∗^*p* < 0.05, ^∗∗^*p* < 0.01, ^∗∗∗^*p* < 0.001, and ^∗∗∗∗^*p* < 0.0001, by two-way ANOVA. (d) Relative quantification of P*memo-1*::GFP of worm treatment with EGCG at day 2 and day 4. ^∗^*p* < 0.05, ^∗∗^*p* < 0.01, ^∗∗∗^*p* < 0.001, and ^∗∗∗∗^*p* < 0.0001, by two-way ANOVA. (e) Representative images of P*memo-1*::GFP reporter worms treated with EGCG at day 2 and day 4, showing quality of MOME-1 following, Scale bar: 200 *μ*m. (f) Estimation body size per *memo-1(gk345)* mutants in treated with EGCG, three replicate experiments; *n* ≥ 6 worms. ^∗^*p* < 0.05, ^∗∗^*p* < 0.01, and ^∗∗∗^*p* < 0.001, by two-way ANOVA. (g) Representative images of *memo-1(gk345)* mutants treated with EGCG at day 2 and day 4. Scale bar: 200 *μ*m.

In addition to mitochondria, NADPH oxidase-generated ROS also acting as a second messenger has been shown to promote both stress resistance and longevity [41]. Moreover, BLI-3/NADPH oxidase is required for physiological dose of EGCG to extend lifespan (Figure 4(b)). To verify whether EGCG initiates health protection mechanisms through NADPH oxidase-mediated redox feedback loops, we tested the effects of EGCG on MEMO-1, the key redox protein of this feedback loop by fluorescence imaging *in vivo*. In larval development, we found that EGCG caused a significant decrease in MEMO-1::GFP along with induced body size reduction of this transgenic strain in a dose-dependent manner (Figures S3a and S3b). Interestingly, we observed a significant increase in the body size and protein levels of GFP::MEMO-1 in day 2 and day 4 of adults subjected to physiological dose EGCG recovery treatment relative to the continuous high dose stress treatment group, where the MEMO-1 protein levels were elevated to a similar level of control (Figures 4(d) and 4(e)). To examine whether MEMO-1 function is required for the health protection mechanism of EGCG, we next used the *memo-1*(gk345) putative null mutants (*memo*-1(-)) and measured physiological health indicators. Under normal conditions, loss of *memo*-1 gene, the *C. elegans* did show lifespan increased compared with *wild-type* animals (Figure 4(a)). By contrast, the health-protective effect of the physiological dose was not reflected in the recovery of body size reduction of mutant induced by the high-dose like the *wild-type* (Figure 4(b) and Figure S3c).

Taken together, these findings demonstrated that NADPH oxidase-induced redox signaling constituting a protective mechanism was required for a physiological dose of EGCG, which could alleviate the body size-reduction caused by high dose and promote longevity. And MEMO-1 is a critical determinant of this mitigating mechanism.

### 3.5. EGCG May Alleviate the Health Defects by Restoring Mitochondrial Function

**Figure 5:**
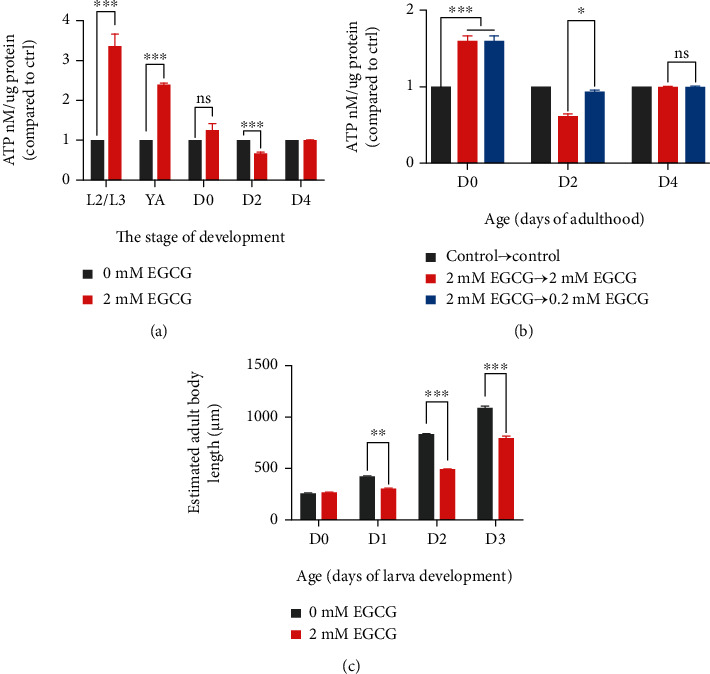
EGCG mitigates and recovers health side effects by restoring mitochondrial function. (a) Relative ATP level treatment with high dose of EGCG during the larval development compared to control. ^∗^*p* < 0.05, ^∗∗^*p* < 0.01, and ^∗∗∗^*p* < 0.001, by two-way ANOVA. (b) Relative ATP level treatment with high dose of EGCG at day 0 and day 2 and maintained it at day 4 of adulthood. Compared to control, ^∗^*p* < 0.05, ^∗∗^*p* < 0.01, and ^∗∗∗^*p* < 0.001, by two-way ANOVA. (c) Estimation body length per worm during the larva development; three replicate experiments; *n* ≥ 6 worms. ^∗^*p* < 0.05, ^∗∗^*p* < 0.01, and ^∗∗∗^*p* < 0.001, by two-way ANOVA.

Mitochondrial function has been shown to be involved in the prolongation of healthy lifespan by EGCG during early-to-mid adulthood in *C. elegans* [8], we next analyzed whether the mitochondrial function determined as ATP produced participated in the health protection mechanism of EGCG in *C. elegans*. We found that the intervention of high doses of EGCG at the onset of larval development caused a significant reduction in body size, conversely causing a significant increase in ATP levels, with a decrease not occurring until the 2th day of adulthood (Figure 5(a)). After 24 hours of palliative treatment with physiological doses of EGCG, the ATP levels of poststress larvae were found to a moderate rebound, but less than that of the control (Figure 5(b)). Thus, these data suggested that excess production of ATP induced by high dose of EGCG was associated with body size and behavioral rate reduction during larval development, while the impaired ATP synthesis caused by high doses during aging is restored after performing physiological dose relief to ensure healthy.

## 4. Discussion

In this study, we found that physiological doses of EGCG could attenuate the health defects of high dose. Mechanistically, we demonstrated a health-protective mechanism of a physiological dose of EGCG that can mitigate the health side effects caused by high-dose exposure by inhibiting the negative regulator MEMO-1 to maintain the feedback loop of the NADPH oxidase-mediated redox signaling.

### 4.1. EGCG Does Not Rely Solely on Its Own Chemical Properties of Antioxidant or Prooxidant to Repair Health Defects

The ability of physiological doses EGCG to prolong life span has been reported for a long time as an antioxidant or prooxidant. But it is different from the previous studies. In this paper, the health side effects of high doses are rescued from physiological dose-mediated protection mechanism, such as prolonged lifespan, increased body size, improved body movement, and a moderate increase in pharyngeal pumping rate. Similarly, adaptive stress caused by physiological doses during larval development can improve resistance to excessive stressful environments in adulthood. Furthermore, EGCG promotes mitochondrial ROS formation and thus leads to the mitochondrial dysfunction. Low or acute mitochondrial ROS levels at the physiological level can act as a second messenger to trigger the recovery by activating the mitohormesis. In addition, the body size of *C. elegans* with physiological doses similarly showed a little reduction. It may be in line with the “Disposable Soma Theory,” which is proposed to reduce body size and promote lifespan by modulating an energy-intensive stress response and repair system [4]. This mechanism needs to be further studied in the future.

We conclude that EGCG does not rely solely on its own chemical properties of antioxidant or prooxidant to repair health defects. It might exhibit either stress system activator in the larval stage to improve the stress resistance of the adult stage at high dose of EGCG or antioxidant in the adult stage to alleviate the oxidative stress damage of high dose of EGCG in the larval stage, which depends on the stress situations, lifecycle, and the concentration of EGCG.

### 4.2. EGCG Requires MEMO-1 Protein to Attenuate the Health Deficiencies

BLI-3/DUXOs, as one kind of the NADPH oxidases, can be activated by generating ROS to adapt to oxidative stress and reestablished cellular homeostasis by a negative regulator MEMO-1 [38, 42]. It was downregulated by EGCG resulting in body size reduction in a dose-dependent manner during larval development. And *memo-1* gene downregulation has been shown to induce ROS production in *C. elegans* that might have longevity-promoting activities analogously to mitochondrially derived ROS [38]. Chronic high dose of EGCG treatment could further suppress [P*memo-1*::GFP] expression and accelerate mitochondrial oxidative stress, whereas physiological doses EGCG can partially restore its expression and are accompanied by a decrease in mitochondrial ROS. The health protection mechanism of physiological dose is precisely through enhancing protein activity of MEMO-1 to reduce high-dose mitochondrial ROS to enhance a hormetic adaptation to mild stress. In contrast, this protective mechanism will fail when MEMO-1 was completely knocked out, and high doses of EGCG induce further body size reduction. Moreover, excess ROS was shown to be generated by the dual oxidase BLI-3 in *memo*-1(gk345). Based upon our analysis, the prooxidant and antioxidant regulation of EGCG is accomplished indirectly by the upregulation of BLI-3. Growth is severely inhibited when the *memo-1* gene is completely knocked out; we concluded that *memo-1* is essential for health protection mechanism of EGCG, which is the central for physiological dose that allows for a moderate recovery period of expression to activate the detoxification feedback loop.

It was interestingly found that loss of gene bli-3 counteracted the lifespan extension of physiological doses of EGCG, while MEMO-1 protein levels elevated by physiological dose EGCG are beneficial to ameliorate the developmental arrest induced by high dose. This suggested that EGCG-induced body size reduction should be the physiological stress response caused by inhibition of MEMO-1. And the detoxification response plays different roles in lifespan and body size regulation.

### 4.3. Mitochondrial Function Is Required for EGCG to Promote Health during Larval and Adulthood

Mitochondrial and cellular antioxidant enzymes are essential for the maintenance of normal mitochondrial function, especially in highly oxidative tissues. Mitochondrial dysfunction has been shown to be caused by multiple mechanisms such as elevated ROS production, impaired function in electron transport complexes and/or activities, reduced membrane potential and adenosine triphosphate (ATP) production, crista damage, enlarged mitochondria, and lower mitochondrial counts [32, 43]. And our results show that a transient increase in ROS level during larval development was induced. Apparently, it is not conducive to healthy development and longevity. Mitochondrial ATP synthase was demonstrated to control larval development cell nonautonomously in Caenorhabditis elegans. It is essential in all tissues for optimal development, especially the body muscles of larval development from the L3 to the L4 stages, while we found the abnormal phenomenon that the transient increase followed by a gradual decrease in ATP release caused by high-dose EGCG, which was similar to poststress body movement. The mitochondrial respiratory chain defects interfere with the production of a global developmental signal. This suggests that high-dose EGCG may have affected the mitochondrial ATP synthesis function during larval development [44].

Subsequently, physiological doses of EGCG increased ATP levels of post-stress larvae. This reflects that the physiological-dose EGCG could repair the mitochondrial dysfunction caused by excess stress which is the main cause of promoting aging. Thus, EGCG elicited toxicity through a mechanism that involved, at least in part, a redox impairment associated with mitochondrial dysfunction, which has been mitigated by triggering mitochondrial biogenesis depending on its own antioxidant-prooxidant pathway. The mechanisms that EGCG elicit mitochondrial biogenesis remain unknown.

## 5. Conclusions

In summary, this work provides novel insights on the health-protective mechanism of EGCG beyond antioxidant properties. And it is regulated by MEMO-1-mediated NADPH oxidase feedback signal in order to adapt to the environment and promote health. Moreover, it provides a new idea for the development and utilization of dietary polyphenols in tea and a reference for the mitigation and recovery of health side effects.

## Data Availability

Readers may access the data underlying the findings of the study by writing to the corresponding author.

## Supplementary Materials

Supplementary Materials Supplementary Figure S1: physiologic doses of EGCG improves body movement of high dose. The number of thrashes of adults from development onset intervention with EGCG per minute in liquid. Three replicate experiments; *n* ≥ 10 worms. ^∗^*p* < 0.05, ^∗∗^*p* < 0.01, and ^∗∗∗^*p* < 0.001, by two-way ANOVA. Supplementary Figure S2: EGCG promotes the formation of ROS during larval development. a Effect of EGCG (2 mM) on O_2_^–^ production during larval development measured by DHE staining. Scale bar: 48 h—100 *μ*m; 72 h—200 *μ*m. b Relative quantification of O_2_^–^ treatment with EGCG at 48 h and 72 h of larval development. ^∗^*p* < 0.05, by two-way ANOVA. Supplementary Figure S3: EGCG inhibited the MOME-1 expression during larval development. a,c Estimation body size of QZ50[Pmemo-1::GFP] mutants treated with EGCG during larval development; three replicate experiments; *n* ≥ 6 worms. ^∗^*p* < 0.05 and ^∗∗^*p* < 0.01, by two-way ANOVA. b,d Relative quantification of Pmemo-1::GFP treatment with EGCG at day 0 of adulthood. ^∗∗∗^*p* < 0.001 and ^∗∗∗∗^*p* < 0.0001, by two-way ANOVA. Scale bar: 200 *μ*m. ii, iii compared with i, images are representative of the expression distribution of Pmemo-1::GFP. *n* ≥ 5 analyzed per treatment, visualized via confocal microscopy. Scale bar: 100 *μ*m.
