# Supplementary material for: Physiological Dose of EGCG Attenuates the Health Defects of High Dose by Regulating MEMO-1 in Caenorhabditis elegans
Source: Oxid Med Cell Longev. 2021 Jun 24;2021:5546493. doi: 10.1155/2021/5546493 (PMC8249131; doi:10.1155/2021/5546493)
Supplement: Supplementary Materials — Supplementary Figure S1: physiologic doses of EGCG improves body movement of high dose. The number of thrashes of adults from development onset intervention with EGCG per minute in liquid. Three replicate experiments; n ≥ 10 worms. ∗p < 0.05, ∗∗p < 0.01, and ∗∗∗p < 0.001, by two-way ANOVA. Supplementary Figure S2: EGCG promotes the formation of ROS during larval development. a Effect of EGCG (2 mM) on O2– production during larval development measured by DHE staining. Scale bar: 48 h—100 μm; 72 h—200 μm. b Relative quantification of O2– treatment with EGCG at 48 h and 72 h of larval development. ∗p < 0.05, by two-way ANOVA. Supplementary Figure S3: EGCG inhibited the MOME-1 expression during larval development. a,c Estimation body size of QZ50[Pmemo-1::GFP] mutants treated with EGCG during larval development; three replicate experiments; n ≥ 6 worms. ∗p < 0.05 and ∗∗p < 0.01, by two-way ANOVA. b,d Relative quantification of Pmemo-1::GFP treatment with EGCG at day 0 of adulthood. ∗∗∗p < 0.001 and ∗∗∗∗p < 0.0001, by two-way ANOVA. Scale bar: 200 μm. ii, iii compared with i, images are representative of the expression distribution of Pmemo-1::GFP. n ≥ 5 analyzed per treatment, visualized via confocal microscopy. Scale bar: 100 μm. [file 5546493.f1.docx]

## Supplementary Materials

**Figure. S1 Physiologic doses of EGCG improves body movement of high dose**


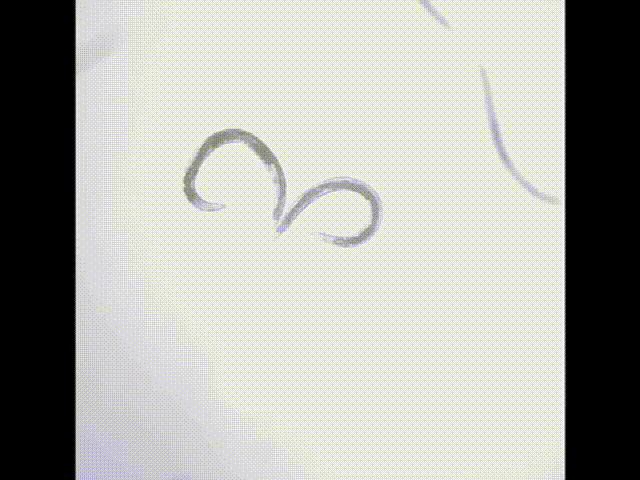

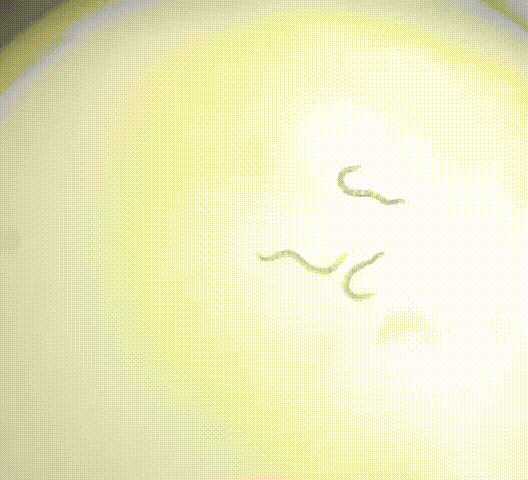

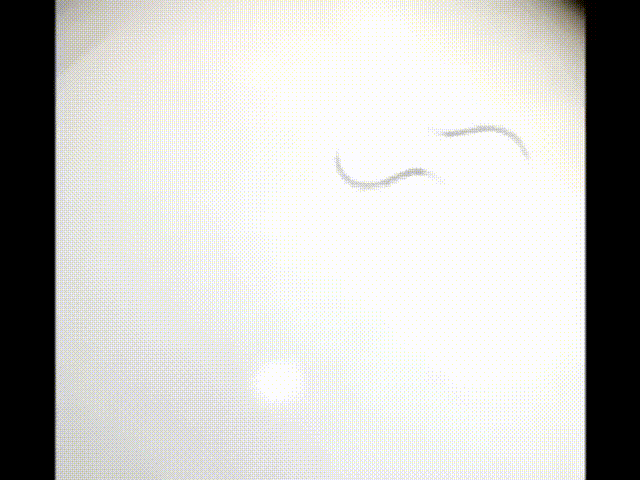

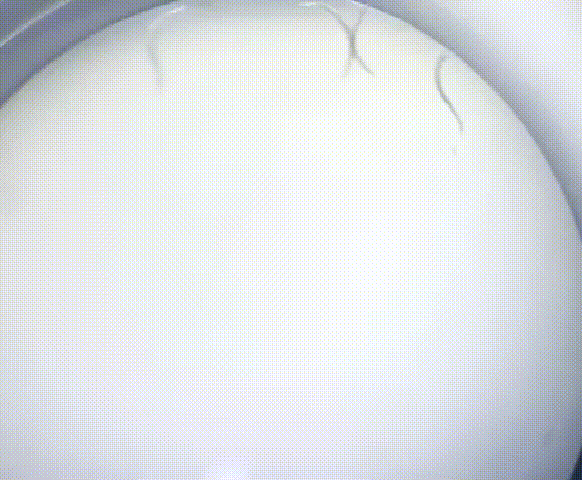


2 mM EGCG→2 mM EGCG

2 mM EGCG→0.2 mM EGCG

0.2 mM EGCG→2 mM EGCG

Control→Control

The body movement of Adults from development onset intervention

Day 6 of adulthood

Number of thrashes per minute in liquid. three replicate experiments; n ≥ 10 worms *p < 0.05, 3 **p < 0.01,***p < 0.001, by two-way ANOVA

**Figure. S2 EGCG promotes the formation of ROS during larval development**


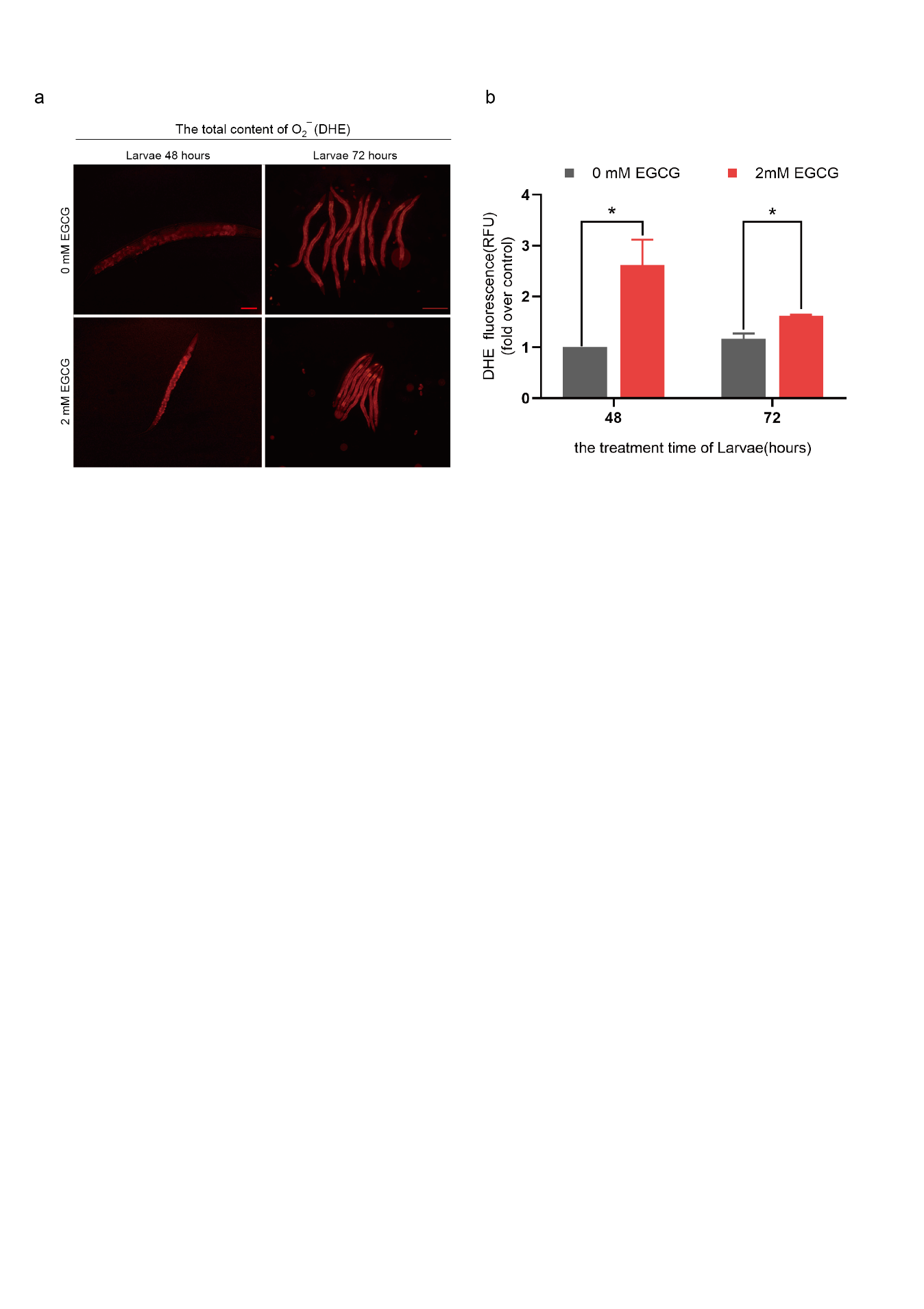


**a** Effect of EGCG (2 mM) on O_2_ ^–^ production during larval development measured by DHE staining. Scale bar: (48h), 100 μm; (72h), 200 μm.

**b** Relative quantification of O_2_ ^–^ treatment with EGCG at 48h and 72h of larval development, *p < 0.05, by two-way ANOVA

**Figure. S3 EGCG inhibites the MOME-1** **expression during larval development**


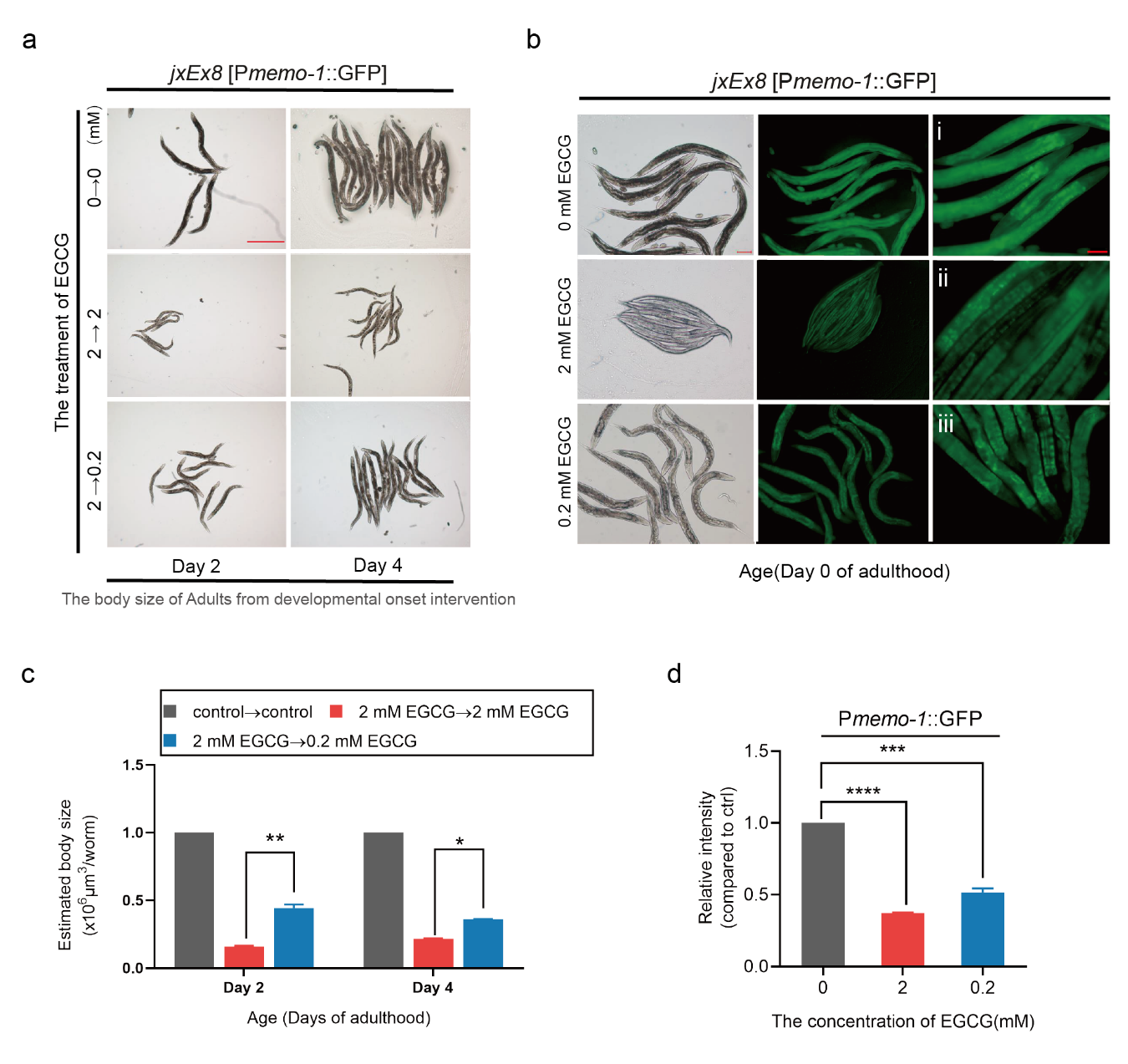


**a,c** Estimation body size of QZ50[P*memo-1*::GFP] mutants treated with EGCG during larval development, three replicate experiments; n ≥ 6 worms *p < 0.05, **p < 0.01, by two-way ANOVA.

**b,d** Relative quantification of Pmemo-1::GFP treatment with EGCG at Day0 of adulthood , ***p < 0.001, ****p < 0.0001, by two-way ANOVA. Scale bar: 200 μm.

**ii, iii** compared with **i,** images are representative of the expression distribution of P*memo-1*::GFP, n ≥ 5 analyzed per treatment, visualized via confocal microscopy. Scale bar :100 μm.
